# Management of patients with fibrosing interstitial lung diseases

**DOI:** 10.1093/ajhp/zxab375

**Published:** 2021-10-05

**Authors:** Lee E Morrow, Daniel Hilleman, Mark A Malesker

**Affiliations:** 1 Creighton University School of Medicine, Omaha, NE; 2 Creighton University School of Pharmacy and Health Professions, Omaha, NE, USA; 3 Creighton University School of Pharmacy and Health Professions, Omaha, NE; 4 Creighton University School of Medicine, Omaha, NE, USA

**Keywords:** drug therapy, idiopathic pulmonary fibrosis, interstitial lung disease, nintedanib, pirfenidone

## Abstract

**Purpose:**

This article summarizes the appropriate use and pharmacology of treatments for fibrosing interstitial lung diseases, with a specific focus on the antifibrotic agents nintedanib and pirfenidone.

**Summary:**

The interstitial lung diseases are a heterogenous group of parenchymal lung disorders with a common feature—infiltration of the interstitial space with derangement of the normal capillary-alveolar anatomy. Diseases characterized by fibrosis of the interstitial space are referred to as the fibrosing interstitial lung diseases and often show progression over time: idiopathic pulmonary fibrosis is the most common fibrotic interstitial lung disease. Historically, therapies for fibrosing lung diseases have been limited in number, questionable in efficacy, and associated with potential harms. Food and Drug Administration (FDA) approval of the antifibrotic agents nintedanib and pirfenidone for idiopathic pulmonary fibrosis in 2014 heralded an era of reorganization of therapy for the fibrotic interstitial lung diseases. Subsequent investigations have led to FDA approval of nintedanib for systemic sclerosis–associated interstitial lung disease and interstitial lung diseases with a progressive phenotype. Although supportive care and pulmonary rehabilitation should be provided to all patients, the role(s) of immunomodulators and/or immune suppressing agents vary by the underlying disease state. Several agents previously used to treat fibrotic lung diseases (*N*-acetylcysteine, anticoagulation, and pulmonary vasodilators) lack efficacy or cause harm.

**Conclusion:**

With the introduction of effective pharmacotherapy for fibrosing interstitial lung disease, pharmacists have an increasingly important role in the interdisciplinary team managing these patients.

**KEY POINTS**

- Although the treatment of fibrotic interstitial lung diseases has historically been empiric—and mostly unsuccessful—nintedanib and pirfenidone are now FDA-approved antifibrotic therapies, with robust data supporting their incorporation into a multifaceted treatment plan.
- Nintedanib and pirfenidone are generally well tolerated, but their prescription is nuanced and requires understanding of the relative advantages and disadvantages of each agent.
- Pharmacists play a key role in caring for these patients, including providing education on polypharmacy and potential adverse effects of medications, advising on prevention and management of adverse effects, and minimizing noncompliance.

The interstitial lung diseases (ILDs) encompass a diverse group of parenchymal lung disorders that involve the pulmonary interstitial space and derange normal capillary-alveolar anatomy. Diseases causing fibrosis to infiltrate the interstitial space are specifically referred to as fibrosing ILDs. Some ILDs have known etiologies, including exposure to antigens like mold or feathers (hypersensitivity pneumonitis), exposure to environmental toxins (eg, asbestosis), or inflammation caused by connective tissue diseases (CTDs) such as rheumatoid arthritis or systemic sclerosis (SSc).^1-5^ Other ILDs, the idiopathic interstitial pneumonias, have no known cause.^6^

Idiopathic pulmonary fibrosis (IPF) is a chronic, progressive idiopathic interstitial pneumonia that occurs mainly in individuals more than 60 years of age, has histopathologic and radiographic patterns of usual interstitial pneumonia, and is associated with an unfavorable prognosis.^7^ Some patients with other fibrosing ILDs unpredictably develop progressive fibrosis (PF-ILD), resulting in poorer outcomes.^8^ Accordingly, patients with fibrosing ILD must be monitored to identify individuals with the PF-ILD phenotype early, as timely initiation of therapies can slow disease progression and improve outcomes.

Because there are limited data focused on PF-ILD, and because IPF is the most common PF-ILD, our current therapeutic strategy for PF-ILD often mirrors that for IPF. In this article we review the appropriate use of treatments for select fibrosing ILDs, including the PF-ILD phenotype.

## Literature search methodology

PubMed was searched for references using the Medical Subject Headings terms “Pulmonary Fibrosis” and “Lung Diseases, Interstitial”. Each search was restricted to clinical trials, meta-analyses, randomized controlled trials, and systematic reviews published in English in the preceding 10 years. Electronic abstracts of identified articles were reviewed for relevance to the treatment of ILD; reference lists of those papers were reviewed to identify additional relevant articles.

## Clinical course and monitoring of fibrosing ILDs

Although the diagnostic algorithm for ILD is beyond the scope of this review, evaluation combines a detailed history, physical examination, pulmonary function testing, radiologic imaging, serologic evaluation, and possibly tissue biopsy. High-resolution computed tomography is essential in the diagnosis of ILDs^7,9^ and can be diagnostic of select entities. However, definitive diagnosis is often challenging despite intensive investigation, and interdisciplinary discussion of such cases is encouraged. Despite use of this best practice, some ILDs remain unclassifiable.^6^

Assessing disease severity and progression in fibrotic ILDs is controversial.^10^ Because declines in forced vital capacity (FVC) of 5% to 10% over 6 to 12 months are predictive of mortality—and FVC is a reproducible measure—the rate of FVC decline is the Food and Drug Administration’s (FDA’s) preferred surrogate measure for death in large therapeutic trials.^11,12^

## Treatment guidelines: Supportive care

Multidisciplinary, collaborative care of patients with IPF is associated with improved quality of care and reduced expenses and is often utilized in other fibrosing ILDs.^13^ Supportive care should be provided to patients with fibrosing ILDs throughout the course of their disease and is not limited to end-of-life situations.^14,15^ The type(s) and timing of supportive care should be individualized to the needs of the patient and their caregivers. General health measures including vaccinations, smoking cessation, and appropriate use of face masks should be encouraged in patients with fibrosing ILDs. Symptom management, supplemental oxygen, pulmonary rehabilitation, and patient education are important elements of the overall care of patients with fibrosing ILDs.^16-19^ Exertional dyspnea and cough are the 2 most common symptoms of fibrosing ILDs.^16^ Exertional dyspnea is typically managed with supplemental oxygen and/or pulmonary rehabilitation. Prescription of supplemental oxygen therapy to patients with fibrosing ILD is poorly studied but is generally provided, consistent with Centers for Medicare and Medicaid Services guidelines.^20^ The use of supplemental oxygen in patients with ILD who are normoxemic at rest but desaturate during exercise is controversial, given the paucity of data specific to this subpopulation.^20,21^ Pulmonary rehabilitation is recommended for all patients with ILD, as compliance with a supervised rehabilitation program improves quality of life (QOL), subjective dyspnea, and exercise tolerance.^22^

Cough in patients with fibrosing ILD is often unresponsive to antitussive drugs. The American College of Chest Physicians has published evidence-based guidelines for therapy of ILD-associated cough.^23^ Because corticosteroids (oral or inhaled) have not been shown to be effective for this indication and predispose to opportunistic infections that can paradoxically increase cough, their use is discouraged.^23^ Thalidomide can suppress cough in patients with fibrosing ILD, but 80% of patients discontinue therapy due to adverse effects (constipation, dizziness, and malaise).^24^ Gabapentin suppresses cough secondary to sensory neuropathic causes and may be considered in patients with fibrosing ILDs.^25^ Opioids may also be considered for refractory cough as the underlying disease progresses.

Fibrosing ILDs are frequently associated with anxiety and depression.^26,27^ Psychosocial support as a component of pulmonary rehabilitation, as well as the use of supplemental oxygen, may improve psychiatric symptoms in these patients.^26^ Pharmacologic and behavioral cognitive management of depression and anxiety should be considered as part of a comprehensive strategy to improve QOL.^27^ Because depression can exacerbate the weight loss seen in advanced pulmonary disease, including the fibrosing ILDs, nutritional support should be part of the pulmonary rehabilitation program.^28,29^

Gastroesophageal reflux disease (GERD) is common in patients with fibrosing ILDs, and microaspiration of acid may contribute to disease progression.^16^ However, results of post hoc subgroup analyses of randomized trials involving patients with IPF are inconclusive concerning the benefit of acid suppressant drug therapy.^30,31^ One multicenter randomized trial found reduced IPF progression and fewer hospitalizations, exacerbations, and deaths 48 weeks after patients with GERD underwent laparoscopic fundoplication.^32^ The small number of patients in this study (*n* = 29 per arm) limits definitive conclusions.

## Treatment guidelines: Pharmacotherapy for IPF

Because IPF was originally considered a chronic inflammatory disorder wherein inflammation caused alveolar fibrosis, early therapy was empiric and immunosuppressing agents (ie, corticosteroids, cyclosporine, azathioprine) were commonly used.^16^ Subsequent observational studies of these agents produced mixed results.^16^ Given poor clinical outcomes with immunosuppression (disease progression, opportunistic infections, and mortality), other immunomodulators (ie, interferon, etanercept, imatinib, *N*-acetylcysteine [NAC]), anticoagulants (warfarin), and pulmonary vasodilators for secondary pulmonary hypertension (ie, endothelin receptor antagonists, sildenafil) were prescribed off-label.^16^ With more rigorous investigations, therapy of IPF has evolved; current recommendations for IPF pharmacotherapy are summarized in Box 1.

Box 1. **Pharmacotherapy for IPF^32,94^**

**Potentially harmful therapies**

Ambrisentan

Everolimus

Prednisolone + azathioprine +

*N*-acetylcysteine combination therapy

Warfarin

**Potentially ineffective therapies**

Bosentan

Imatinib

Macitentan

*N*-acetylcysteine

Sildenafil

**Effective disease-modifying therapies**

Antacid therapy

Nintedanib

Pirfenidone

### Immunomodulators

 In one of the first randomized trials of pharmacotherapy in IPF, interferon γ-1b (IFN) combined with prednisolone was compared to prednisolone alone in 18 patients at a single center in Austria. Significant improvements in total lung capacity and arterial oxygen saturations were seen during 12 months of open-label therapy with IFN.^34^ These promising results led to a larger study of IFN in 330 patients with IPF from 58 centers in the United States, Europe, Canada, and South Africa.^35^ This study randomized patients to IFN (200 µg subcutaneously 3 times weekly, *n* = 162) or placebo (*n* = 68) for 52 weeks. Although IFN did not improve the primary endpoint of progression-free survival, all-cause mortality was 10% with IFN and 17% with placebo (*P* = 0.08). An exploratory analysis found survival was improved in IFN-treated patients with less severe impairment in baseline FVC even though the drug did not slow the rate of FVC decline.

These results led to the INSPIRE (International study of Survival outcomes in idiopathic Pulmonary fibrosis with InteRfEron gamma-1b) study, an international trial to assess the effect of IFN on all-cause mortality in IPF. The INSPIRE trial randomized patients with an FVC of ≥55% and a carbon monoxide diffusing capacity (Dlco) of ≥35% to IFN (200 µg subcutaneously 3 times weekly, *n* = 551) or placebo (*n* = 275).^36^ The study was stopped for futility after a mean duration of therapy of 64 weeks, with mortality rates of 15% and 13% in patients treated with IFN and placebo, respectively. IFN had no significant effect on the rate of decline in FVC compared to placebo. This trial provided convincing evidence that IFN therapy is not beneficial in IPF.

Trials of the immunomodulating drugs etanercept (a tumor necrosis factor inhibitor) and imatinib (a tyrosine kinase inhibitor) have also failed to demonstrate benefit in IPF, as assessed using serial lung function and subjective disease progression as outcomes.^37,38^

### Immune-suppressing agents

 One of the most recent trials of immunosuppressive therapy in IPF, the PANTHER-IPF (Prednisone, Azathioprine, *N*-Acetylcysteine: A Study That Evaluates Response in Idiopathic Pulmonary Fibrosis) trial, demonstrated overt harm with immune suppression. This double-blind study randomized patients with IPF to one of 3 groups: (1) three-drug (prednisone, azathioprine, and NAC) therapy; (2) NAC monotherapy; and (3) triple placebo. At an interim analysis with approximately 75 patients randomized to each group, the 3-drug combination therapy arm had a 10% mortality rate, compared to a 1% mortality rate in the triple placebo arm.^39^ Hospitalizations and serious adverse events were also significantly increased with 3-drug therapy, and this arm of the trial was terminated early.

Unlike in IPF, immunosuppressants are widely used to treat patients with CTD-associated ILDs and hypersensitivity pneumonitis.^40,41^ Immunosuppressants are also widely used to treat nonpulmonary manifestations of CTDs. Although immunosuppression slows the progression of SSc-associated ILD,^42,43^ the evidence that immunosuppressants slow the progression of fibrosing ILDs other than SSc-associated ILD is limited.

### *N*-acetylcysteine

 After the 3-drug arm of the PANTHER-IPF study was discontinued, enrollment in the NAC monotherapy arm of the study continued. However, after 60 weeks of treatment, there were no significant differences in rates of decline of FVC, mortality, or acute exacerbations between NAC- (*n* = 133) and placebo-treated patients (*n* = 131).^44^ A subsequent small, randomized, double-blind trial suggested the combination of NAC and pirfenidone was associated with an increase in the rate of lung function decline compared to pirfenidone alone^45^ Collectively, these studies did not support the use of NAC in IPF.

### Endothelin receptor antagonists

The endothelin receptor antagonists (ERAs) have been studied as antifibrotic agents in randomized controlled trials in patients with IPF. Endothelin is a vasoconstrictor that increases inflammation, fibrosis, and endothelial dysfunction in patients with pulmonary hypertension. Although poorly understood in the pathophysiology of IPF, elevated serum levels of endothelin and increased expression of endothelin receptors in lung tissue have been observed in patients with IPF.^46^

The first controlled trial of an ERA as an antifibrotic in IPF randomized 74 patients to bosentan and 84 patients to placebo.^47^ Although bosentan failed to improve 6-minute walk distance (6MWD) or slow the decline in FVC, bosentan use was demonstrated to be associated with favorable trends in time to death, QOL, and disease progression (*P* = 0.12). In a subgroup analysis of patients with biopsy-proven IPF, a statistically significant reduction in disease progression was found in bosentan-treated patients (*P* = 0.009). In a subsequent trial involving patients with biopsy-proven IPF of less than 3 years’ duration, bosentan-treated patients (*n* = 402) did not show improvements in time to worsening (ie, a decline in FVC or Dlco), in QOL, or in dyspnea scores when compared to placebo recipients (*n* = 409).^48^

Two randomized trials using different ERAs (ambrisentan and macitentan) as antifibrotic agents also failed to demonstrate improvements in disease progression in IPF.^49,50^ Trends toward an increased risk of disease progression, hospitalizations, and death were seen in the study with ambrisentan.^49^ The reason for these divergent results within the ERA drug class—trends toward benefit with bosentan and trends toward harm with ambrisentan and macitentan—is unknown but may relate to differences in endothelin receptor activity, varying study designs, or other factors. Regardless, the ERAs are not FDA approved for any ILD-related indication and should not be used when managing patients with ILD.

### Anticoagulation

 Anecdotal evidence suggests that patients with IPF may have an increased risk of thromboembolic events.^51^ A randomized, placebo-controlled trial of warfarin (titrated to achieve an international normalized ratio [INR] of 2.0-3.0) was conducted in patients with IPF without another indication for anticoagulant or antiplatelet therapy.^52^ An interim analysis conducted 15 months after study initiation found a significant increase in all-cause mortality in the warfarin group (19.4%) versus the placebo group (4.1%). Although the majority of deaths were attributed to respiratory causes—no cases of fatal bleeding were seen—warfarin was not associated with any significant effect on indices of lung function (FVC, Dlco, and 6MWD). Accordingly, routine use of anticoagulation in patients with IPF does not appear to be beneficial and may be harmful.

### Antifibrotic agents

Given the limitations of the therapies discussed thus far, clinicians caring for patients with PF-ILD have increasingly called for effective therapies specifically targeting pulmonary parenchymal fibrosis. Two agents—nintedanib and pirfenidone—were approved by FDA in 2014 after their efficacy and safety in the treatment of IPF were demonstrated.^53-55^ Both agents provide similar clinical benefit (in terms of rate of FVC decline over time), and no data are available to guide the clinician in choosing one agent over another. Accordingly, the choice between these agents is usually individualized based on each patient’s preference regarding dosing, the most likely adverse effects of each agent, and, given their substantial cost, insurance coverage.^56^ The pharmacology of these agents is summarized in Table 1.

**Table 1.** Pharmacology and Pharmacokinetics of Nintedanib and Pirfenidone^52-55^

	Nintedanib	Pirfenidone
Mechanism of action	Tyrosine kinase inhibition	Inhibition of TGF-β production
Efficacy	Slows FVC decline by 50%	Slows FVC decline by 50%
FDA-approved dosage for IPF	150 mg b.i.d., doses 12 hours apart, with food	801 mg t.i.d., with food
Dosage forms	100- and 150-mg capsules	267- and 801-mg capsules
*t*_max_	4 hours (with food)	3 hours (with food)
Metabolism	Hydrolysis via esterases, then glucuronidation via UGT1A1, 1A7, 1A8, and 1A10; metabolized to minor extent via CYP3A4	Hepatic CYP1A2; metabolized to lesser extent via CYP2C9, 2C19, 2D6, 2E1
Elimination	Feces (93.4%)	Renal as 5-carboxy metabolite (80%)
*t*_½_ (terminal)	9.5 hours	3 hours
Drug-drug interactions	Concurrent use of P-gp and CYP3A4 inhibitors may increase nintedanib exposure	CYP1A2 inhibitors can increase pirfenidone levels; CYP1A2 inducers can reduce pirfenidone levels
Common adverse effect	Diarrhea	Anorexia, nausea, photosensitivity
Monitoring	LFTs at baseline and monthly for 3 months, then periodically; pregnancy test at baseline	LFTs at baseline and monthly for the first 6 months, every 3 months thereafter, and as clinically indicated
Hepatic dosing adjustment		
Mild impairment (Child-Pugh class A)	100 mg b.i.d., 12 hours apart, with food	801 mg t.i.d., with food
Moderate impairment (Child-Pugh class B)	Not recommended	801 mg t.i.d., with food
Severe impairment (Child-Pugh class C)	Not recommended	Not recommended
Renal dosing adjustment		
Mild to moderate impairment (CLcr of 30-90 mL/min)	150 mg b.i.d., 12 hours apart, with food	Dosing not studied; use with caution
Severe impairment (CLcr of <30 mL/min)	Dosing not studied; use with caution	Dosing not studied; use with caution
End-stage renal disease	Dosing not studied; use with caution	Not recommended

Abbreviations: CL_cr_, creatinine clearance; FVC, forced vital capacity; LFT, liver function test; P-gp, P-glycoprotein; *t*_½_, half-life; *t*_max_, time to maximum concentration; TGF-β, transforming growth factor β.

Given IPF’s progressive nature and poor prognosis, early diagnosis and prompt treatment with an approved antifibrotic therapy are important in slowing disease progression and improving outcomes.^57-59^ The decisions as to whether to treat patients with other fibrosing ILDs, which treatments to use, and when to escalate treatment should be individualized based on disease severity, disease progression, and the risk:benefit ratio of treatment options.^60,61^

### Nintedanib

Nintedanib (Ofev; Boehringer Ingelheim Pharmaceuticals, Inc.) is an oral tyrosine kinase inhibitor indicated for the treatment of IPF and chronic fibrosing ILDs with a progressive phenotype, and for reducing the rate of FVC decline in patients with SSc-associated ILD.^50^ Nintedanib at a dosage of 150 mg twice daily reduces the rate of decline of FVC in patients with these diseases by about 50%.^62-65^

#### Clinical trial data

Nintedanib was studied in 3 randomized, double-blind, placebo-controlled, 52-week trials in patients with IPF. In a phase 2 investigation (the TOMORROW trial), nintedanib-treated patients had dose-dependent reductions in the rates of lung function decline and acute IPF exacerbations relative to placebo users.^65^ Two phase 3 trials (INPULSIS-1 and INPULSIS-2) compared nintedanib with a placebo in patients with IPF. Compared to placebo use, nintedanib use reduced the rate of FVC decline compared to placebo (–114.7 mL/year vs –239.9 mL/year in INPULSIS-1 and –113.6 mL/year vs –207.3 mL/year in INPULSIS-2).^62^ Diarrhea was the most common adverse effect and led to discontinuation of study medication in less than 5% of patients.^62^

The longer-term efficacy and safety of nintedanib were evaluated in the INPULSIS-ON trial, an open-label extension study wherein participants had a mean duration of exposure to nintedanib of 44.7 months.^66^ As in the TOMORROW and INPULSIS trials, the most common adverse effect was diarrhea. The annual rate of FVC decline seen in the INPULSIS-ON trial was consistent with the rates in the INPULSIS trials.^66^

In a randomized, double-blind, placebo-controlled, phase 3 clinical trial (the INBUILD trial), patients with fibrosing lung diseases other than IPF who met study-specific criteria for progression of ILD were assigned to nintedanib or placebo. The 332 patients treated with nintedanib had a significantly lower rate of decline in FVC when compared to 331 placebo-treated patients (–80.8 mL/year vs –187.8 mL/year, *P* < 0.001), with no evidence of a differential treatment effect among patients with different diagnoses.^67,68^ In the SENSCIS trial, patients with SSc-ILD were randomized to nintedanib or placebo. The 264 nintedanib-patients treated had a significantly lower rate of decline in FVC when compared to 275 placebo-treated patients (–52.4 mL/year vs –93.3 mL/year, *P* = 0.04).^63^

#### Dosing

 The recommended dosage of nintedanib is 150 mg twice daily, with each dose to be taken with food approximately 12 hours apart, and can be continued indefinitely.^54^ The capsules should not be chewed or crushed because of bitter taste. In patients with mild hepatic impairment (Child-Pugh class A), the recommended dosage is 100 mg twice daily.

#### Pharmacokinetics

Nintedanib is a substrate of P-glycoprotein (P-gp) and, to a lesser extent, cytochrome P450 isozyme CYP3A4. Concurrent administration of potent P-gp and CYP3A4 inhibitors (eg, ketoconazole) increases nintedanib exposure by 50% to 60%; patients should be monitored closely for tolerability during concurrent use of the inhibitor.^69,70^ When possible, these and other inducers (eg erythromycin, carbamazepine, phenytoin, rifampin, St. John’s wort) should be avoided in patients taking nintedanib. Because smoking is associated with decreased nintedanib exposure, which might alter the efficacy profile, patients should be counseled to stop smoking prior to treatment.^54^

#### Safety and precautions

Nintedanib is a pregnancy category D medication: women of childbearing age should have a negative pregnancy test prior to initiation and should avoid becoming pregnant while on nintednaib.^54^ Arterial thrombotic events are more common with nintedanib versus placebo use (rates of 2.5% vs 1.0% for all thrombotic events and 1.5% vs 1.0% for myocardial infarction), and patients should be monitored for these events proactively.^54^ The risk of bleeding while on nintedanib was increased relative to the rate with placebo use in IPF and SSc-ILD studies (10% vs 7% in IPF, 11% vs 8% in SSc-ILD) but not in PF-ILD studies (11% vs 13%).^54^ Epistaxis was the most frequent bleeding event, with some events causing death.^54^ Nintedanib may increase the risk of gastrointestinal perforation in IPF relative to placebo (1% vs 0%): no instances of gastrointestinal perforation occurred in the SSc-ILD or PF-ILD studies using nintedanib.^54^

### Pirfenidone

Pirfenidone (Esbriet; Genentech Inc.) is a pyridine derivative with anti-inflammatory, antifibrotic, and antioxidant effects.^71^ Pirfenidone is labeled only for treatment of IPF.^55^

#### Clinical trial data

The use of pirfenidone in IPF is supported by results of 4 randomized, placebo-controlled, clinical trials. A phase 2, proof-of-concept study of 107 Japanese patients was stopped early for favorable efficacy during an interim analysis.^72^ In the subsequent 52-week, phase 3 trial, pirfenidone significantly decreased the rate of FVC decline and increased progression-free survival in Japanese subjects with IPF.^73^ These two studies led to regulatory approval of pirfenidone in Japan for treating IPF.

The CAPACITY trials (studies 004 and 006) were designed to confirm the effect of pirfenidone on the rate of decline in lung function in subjects from Australia, Europe, and North America.^74^ Although study 004 demonstrated a reduced deterioration of lung function over 18 months with use of pirfenidone vs placebo (–8.0% vs –12.4%, *P* = 0.001), study 006 did not demonstrate a meaningful effect of pirfenidone on mean FVC change at 72 weeks (–9.0% vs –9.6%, *P* = 0.50).^74^ After completion of these trials, pirfenidone was licensed in Europe for patients with IPF with mild to moderate disease.^75^ However, FDA did not approve pirfenidone for use, deeming the CAPACITY trials inconclusive.

The ASCEND study randomized 555 patients with IPF to pirfenidone or placebo for 52 weeks. In this trial pirfenidone slowed the rate of FVC decline (mean decline from baseline, –235 mL vs -428 mL with placebo use; *P* < 0.001), reduced the proportion of patients with a drop in their 6MWD of ≥50 m (25.9% vs 35.7%, *P* < 0.04), and increased the rate of progression-free survival (31.8% vs 16.5%, *P* < 0.001).^76^ These results led to FDA approval of pirfenidone for IPF in 2014. The longer-term safety of pirfenidone was demonstrated in an open-label extension study (the RECAP study) in IPF patients who completed the CAPACITY or ASCEND trials.^77^

A recent phase 2 trial studied the efficacy and safety of pirfenidone in patients with progressive unclassifiable ILD.^78^ In this randomized, placebo-controlled trial, patients were randomized to 24 weeks of pirfenidone (*n* = 127) or placebo (*n* = 126). Although intraindividual variability in home spirometry precluded the study’s prespecified analysis, hospital-based spirometry showed a reduction of the absolute FVC loss in the pirfenidone group. Favorable effects on 6MWD and Dlco were also seen. Pirfenidone is not approved for patients with unclassifiable ILD.

#### Dosing

The recommended dosage of pirfenidone is 801 mg 3 times daily, following a 14-day dose titration.^55^ Treatment is initiated at a dosage of 267 mg 3 times daily on days 1 though 7. On day 8, the dosage is escalated to 534 mg 3 times daily, and from day 15 forward the maintenance dosage is 801 mg 3 times daily (2,403 mg/day in total). Pirfenidone should be taken with food to minimize gastrointestinal (GI) complaints. Consideration can be given to dose reduction, treatment interruption, and/or drug discontinuation for management of adverse reactions. If serum alanine aminotransferase (ALT) and/or aspartate aminotransferase (AST) is elevated to more than 3 times but less than 5 times the upper limit of normal and is accompanied by symptoms or hyperbilirubinemia, it is recommended that the dose be reduced. Pirfenidone should be stopped if the ALT and/or AST is greater than 5 times the upper limit of normal and accompanied by symptoms or hyperbilirubinemia.^55^

#### Pharmacokinetics

Pirfenidone is primarily metabolized by CYP1A2 (70%-80%), with minor contributions from CYP2C9, CYP2C19, CYP2D6, and CYP2E1. The concurrent use of pirfenidone with CYP1A2 inducers may decrease exposure and reduce treatment efficacy. The dosage of pirfenidone should be reduced to 267 mg 3 times daily in the presence of strong CYP1A2 inhibitors (eg, fluvoxamine) or to 534 mg 3 times daily in the presence of moderate CYP1A2 inhibitors (eg, ciprofloxacin). Patients who smoke should be counseled on cessation, as decreased pirfenidone exposure has been reported in smokers.^55^

#### Safety and precautions

Pirfenidone has not been assigned to an FDA pregnancy category.^55^ In postmarketing surveillance, serious cases of pirfenidone-induced liver injury, including fatalities, were reported. Accordingly, liver function tests (LFTs) are recommended prior to the initiation of pirfenidone, monthly for the first 6 months, and every 3 months thereafter.^55^ Patients should be counseled on sunlight/sunlamp avoidance, use of sunscreen (with an SPF of ≥50), and protective clothing, as photosensitivity has been noted in up to 9% of pirfenidone-treated patients.^55^

### Combination therapy

The INJOURNEY trial investigated the tolerability, safety, and pharmacokinetics of nintedanib with add-on pirfenidone therapy.^79^ This exploratory, open-label, randomized trial compared combination nintedanib and pirfenidone therapy versus nintedanib monotherapy in 105 patients with IPF. The primary endpoint was the incidence of GI adverse effects from baseline to week 12. GI adverse effects manifested in 70% of patients in the combination therapy group, compared to 53% of those in the nintedanib monotherapy group.^79^ Because complaints were generally manageable, the investigators concluded their results supported further investigation of combination therapy.

The INSTAGE trial was a randomized, placebo-controlled trial of sildenafil plus nintedanib versus nintedanib alone in patients with advanced IPF (Dlco of ≤35% of predicted).^80^ Although no safety concerns were identified in the combination therapy arm, the addition of sildenafil to nintedanib failed to improve QOL at week 12 relative to nintedanib monotherapy. A randomized, placebo-controlled trial of sildenafil plus pirfenidone in patients with advanced IPF (Dlco of <40% of predicted) did not confer a benefit relative to pirfenidone alone for a composite endpoint of disease progression (reduced 6MWD, respiratory-related hospital admission, and all-cause mortality) over 52 weeks.^81^

### Management of common adverse effects of treatments for fibrosing ILDs

 The most common adverse reactions (ie, those with an incidence of >5%) with use of nintedanib include diarrhea, nausea, abdominal pain, vomiting, abnormal LFT results, decreased appetite, headache, weight loss, and hypertension.^54^ In the INPULSIS trials, GI concerns were the most frequent adverse events reported in patients with IPF treated with nintedanib: diarrhea was reported in 62% of subjects (versus 18% of those receiving placebo); nausea, in 24% of subjects; and vomiting, in 12% of subjects.^82^ If GI symptoms do not resolve with supportive therapies (hydration and loperamide), temporary or permanent dosage reduction to 100 mg twice daily or discontinuation of nintedanib may be necessary.^54,83^ Diarrhea leads to dose reduction in 11% of patients and treatment discontinuation in 5% of patients receiving nintedanib.^54^ Because nintedanib therapy may be associated with elevated LFT results (≤6% of patients) and/or bilirubin elevations (≤5% of patients), LFTs should be performed prior to initiation of nintedanib, at regular intervals during the first 3 months of treatment, and then periodically as clinically indicated.^54^ Although increased LFT values are usually reversible with dose reduction or treatment interruption, cases of severe liver injury and death have been reported in the postmarketing period.^54^

The most frequent adverse effects (ie, those occurring at a rate of >10%) with pirfenidone use include nausea, rash, abdominal pain, upper respiratory infection, diarrhea, fatigue, headache, dyspepsia, dizziness, vomiting, anorexia, GERD, sinusitis, insomnia, weight loss, and arthralgia.^55^ Pirfenidone-associated nausea and vomiting can be diminished by the administration of antacids and/or antiemetics.^56^ Dose reductions or treatment interruptions may be needed to manage adverse events.^55,83,84^

Dose adjustments and/or temporary therapeutic interruptions used to manage adverse events in patients treated with antifibrotic therapy do not have a meaningful impact on the rate of lung function decline.^85,86^ However, every attempt to maintain patients on the recommended dosages (nintedanib, 150 mg twice daily; pirfenidone, 801 mg 3 times daily) should be made since they are the most effective for slowing ILD progression.^65,74^

### Summary of pharmacotherapy for fibrotic ILDs

The 2015 ATS/ERS/JRS/ALAT clinical practice recommendations for IPF are included in Box 1.^87^ The guideline development panel conditionally recommended the use of either nintedanib or pirfenidone for the treatment of IPF and, as in the 2011 guideline, also conditionally recommended antacid therapy. The panel conditionally recommended against the use of sildenafil, macitentan, and bosentan. The panel strongly recommended against the use of warfarin, imatinib, ambrisentan, and the combination of prednisone, azathioprine, and NAC.^87^

### Cost-effectiveness

 The costs of nintedanib and pirfenidone each exceed $100,000 annually in the United States.^56^ Although published studies uniformly found these drugs to be cost-effective, these data came from European countries with centralized healthcare.^88-90^ The superiority of one agent relative to the other is unclear: while data from the United Kingdom showed equal cost-effectiveness, French data showed superiority with pirfenidone, and Belgian data showed superiority with nintedanib.^88-90^ The cost-effectiveness of these agents when used in combination is unknown.

### Future directions

 Given the prevalence of ILD, its substantial morbidity and mortality, a limited number of FDA-approved therapies, and substantial cost of care, further study of ILD pharmacotherapy is needed. Head-to-head trials and comparisons of combination therapy with single-drug therapy would clarify what are currently empiric decisions. Examination of ILD subsets (patients with advanced fibrosis or obesity, the extremely aged, etc) is generally lacking. Because experience with antifibrotics is in its infancy, long-term efficacy and mortality data are suboptimal. The use of antifibrotics post lung transplant is intriguing, as fibrosis is a presumed mechanism of chronic allograft rejection. Other investigations (eg, research on use of P2X2/3 receptor antagonists for chronic cough) could offer novel treatments and better control of distressing ILD symptoms.

## Treatment guidelines: Lung transplantation

Among the common indications for lung transplantation, ILD has the worst prognosis and is associated with high waiting list mortality.^91^ Because the observational IPF Prospective Outcomes (IPF-PRO) Registry found that mortality was independently associated with patient-reported increasing symptom burden and reduced tolerance of physical activity, these manifestations should prompt early referral for lung transplant evaluation.^92,93^ Although they are beyond the scope of this review, the International Society for Heart and Lung Transplantation has published guidelines for timing lung transplantation in IPF patients based upon various prognostic indicators.^91^

## Role of the pharmacist

Antifibrotic therapies are usually initiated in the outpatient setting and several are dispensed by specialty pharmacies. As part of the interdisciplinary healthcare team, pharmacists play key roles in managing fibrosing ILDs. These roles include educating patients on the potential adverse effects of their medications, advising on how to prevent and manage adverse effects, and minimizing the risk that patients will discontinue their medication because of them. In addition to ILD-specific treatments, these patients often receive multiple drugs to treat other manifestations of their disease or comorbidities.^94,95^ Accordingly, careful consideration of potential drug-drug interactions is important. Pharmacists also play an important role in providing accurate education on fibrosing ILDs and their treatments, including directing patients to reliable online resources; these include the Pulmonary Fibrosis Foundation and pharmaceutical manufacturer websites.^96^ Given the substantial costs of some therapies, pharmacists should assist patients with prescription prior authorization and access to industry-sponsored financial assistance programs.

## Conclusion

Patients with IPF, and a proportion of patients with other forms of fibrosing ILD, develop progressive fibrosis with reduced FVC, worsening QOL, and early mortality. All patients with IPF should be offered antifibrotic therapy (nintedanib or pirfenidone) to slow disease progression and improve outcomes. Nintedanib has also been approved for treating other chronic fibrosing ILDs with a progressive phenotype and for reducing the rate of FVC decline in patients with SSc-ILD. Most patients with CTD-associated ILDs will be receiving immunosuppressant therapy, either as a treatment for ILD or as a treatment for other manifestations of CTD.

Pharmacists fulfill important roles in managing fibrosing ILDs, including education on the appropriate use of medications and management of therapy adverse effects. In addition to pharmacotherapy, supportive care and patient education regarding the importance of medication adherence to improve long-term outcomes are key aspects of caring for patients with fibrosing ILDs.
